# Case Report: Biatrial Myxoma With Pulmonary Embolism and Cerebral Embolism: Clinical Experience and Literature Review

**DOI:** 10.3389/fcvm.2022.812765

**Published:** 2022-02-02

**Authors:** Haifeng Ran, Guiqin Chen, Jie Hu, Yulun He, Junwei Liu, Fangling Li, Heng Liu, Tijiang Zhang

**Affiliations:** Department of Radiology, The Affiliated Hospital of Zunyi Medical University, Medical Imaging Center of Guizhou Province, Zunyi, China

**Keywords:** biatrial myxoma, pulmonary embolism, cerebral embolism, computed tomography, magnetic resonance imaging, echocardiography

## Abstract

Cardiac myxoma is a common benign primary intracardiac tumor in the general population, and it is generally characterized as a benign tumor, and the morbidity of biatrial myxoma is low. Cases of biatrial myxoma in young patients are extremely rare. Furthermore, severe complications of cardiac myxoma, such as cerebral embolism, can have fatal consequences. Imaging can effectively assist in making a correct diagnosis and a safe and efficient surgical treatment plan. In this case report, we describe a unique case of a young woman who presented with biatrial myxoma accompanied by pulmonary embolism and cerebral embolism. Computed tomography pulmonary angiography (CTPA) detected multiple filling defects in the bilateral cardiac and bilateral inferior pulmonary artery basal branches. Transthoracic echocardiography (TTE) revealed irregular isoechoic masses in the bilateral atrium. Postoperative histopathology confirmed a biatrial myxoma. The patient was discharged on the ninth day after surgery.

## Introduction

Cardiac myxoma is a common benign primary intracardiac tumor with an incidence rate of 0.0017% (1). Atrial myxoma occurs most commonly in middle-aged and older women, and the most frequent site of cardiac myxoma is the left atrium, followed by the right atrium. However, biatrial myxoma is relatively rare, and contributes to only 2.5% of the total incidence of cardiac myxoma (2, 3). In particular, biatrial myxoma complicating pulmonary embolism and cerebral embolism is extremely rare in young patients. To the best of our knowledge, such cases have not yet been reported in the literature. The awareness of embolic events due to atrial myxoma in young patients is still unsystematic and incomplete. When fragments of biatrial myxoma produce complicating pulmonary embolism and cerebral embolism, patients will be in a critical situation, and it is essential to diagnose and treat the patient as soon as possible to stop the aggravation of the disease and save the patient's life. In this article, we describe a rare biatrial myxoma complicated by pulmonary embolism and cerebral embolism, and review its clinical and imaging characteristics reported in previous cases. These characteristics help clinicians and radiologists pay attention to this disease and can effectively assist in establishing accurate diagnosis and developing a safe and efficient surgical treatment plan.

## Case Description

**Figure 1:**
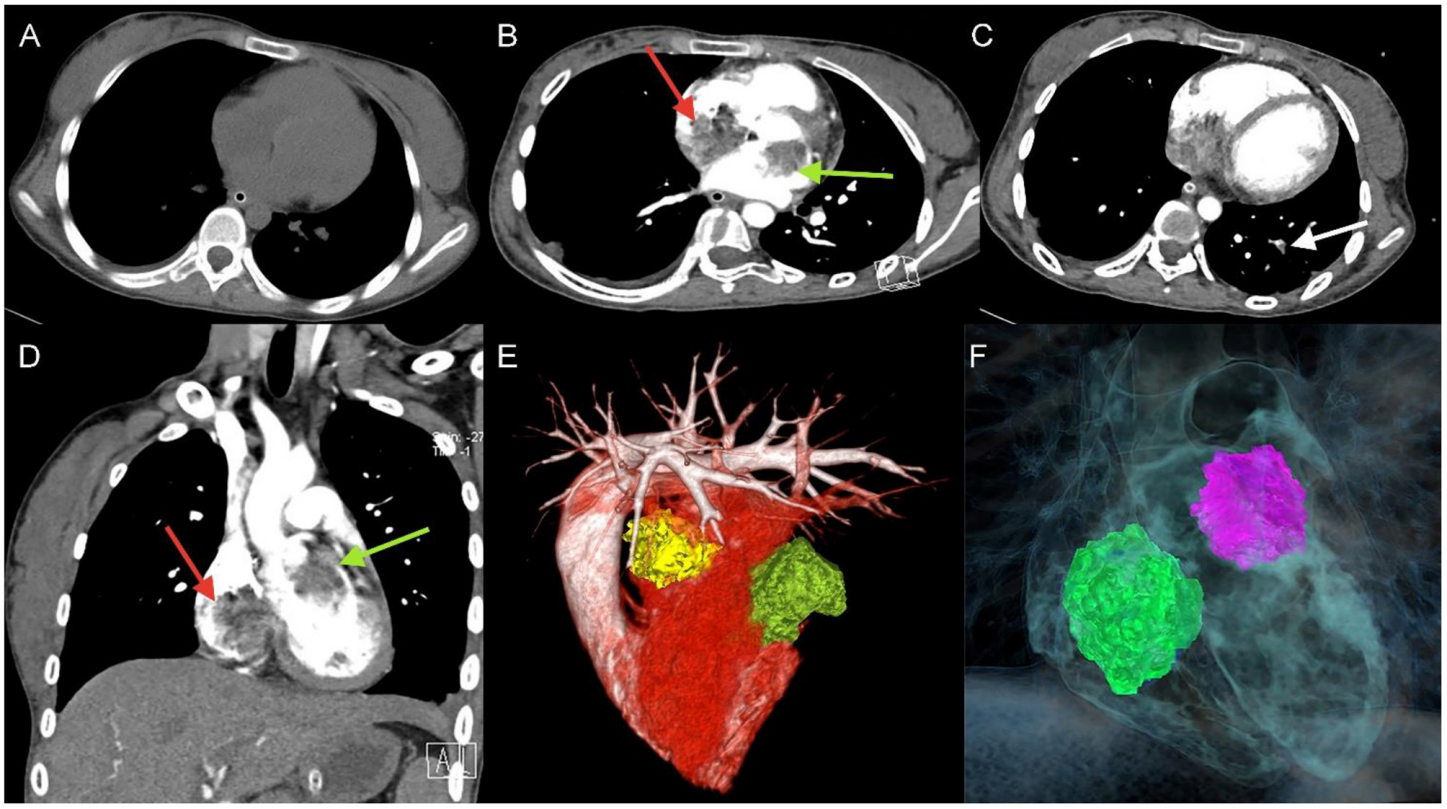
Unenhanced thoracic CT **(A)**, axial and coronal views of Computed tomography pulmonary angiography (CTPA) detected filling defects in right atrium (red arrow), left atrium (green arrow) **(B,D)**, and left lower pulmonary basilar artery (white arrow) **(C)**, three-dimensional reconstruction of heart **(E)** and **(F)** corresponding schematic illustration demonstrating the biatrial myxoma.

**Figure 2:**
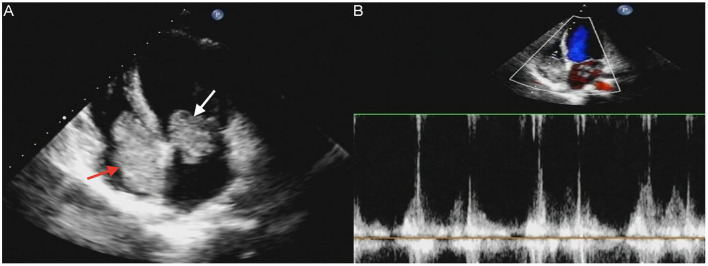
Transthoracic echocardiography (TTE) revealed masses attached to the atrial septum in the left atrial cavity (white arrow) and the right atrial cavity (red arrow) **(A)**. Acceleration of tricuspid valves antegrade flow **(B)**.

**Figure 3:**
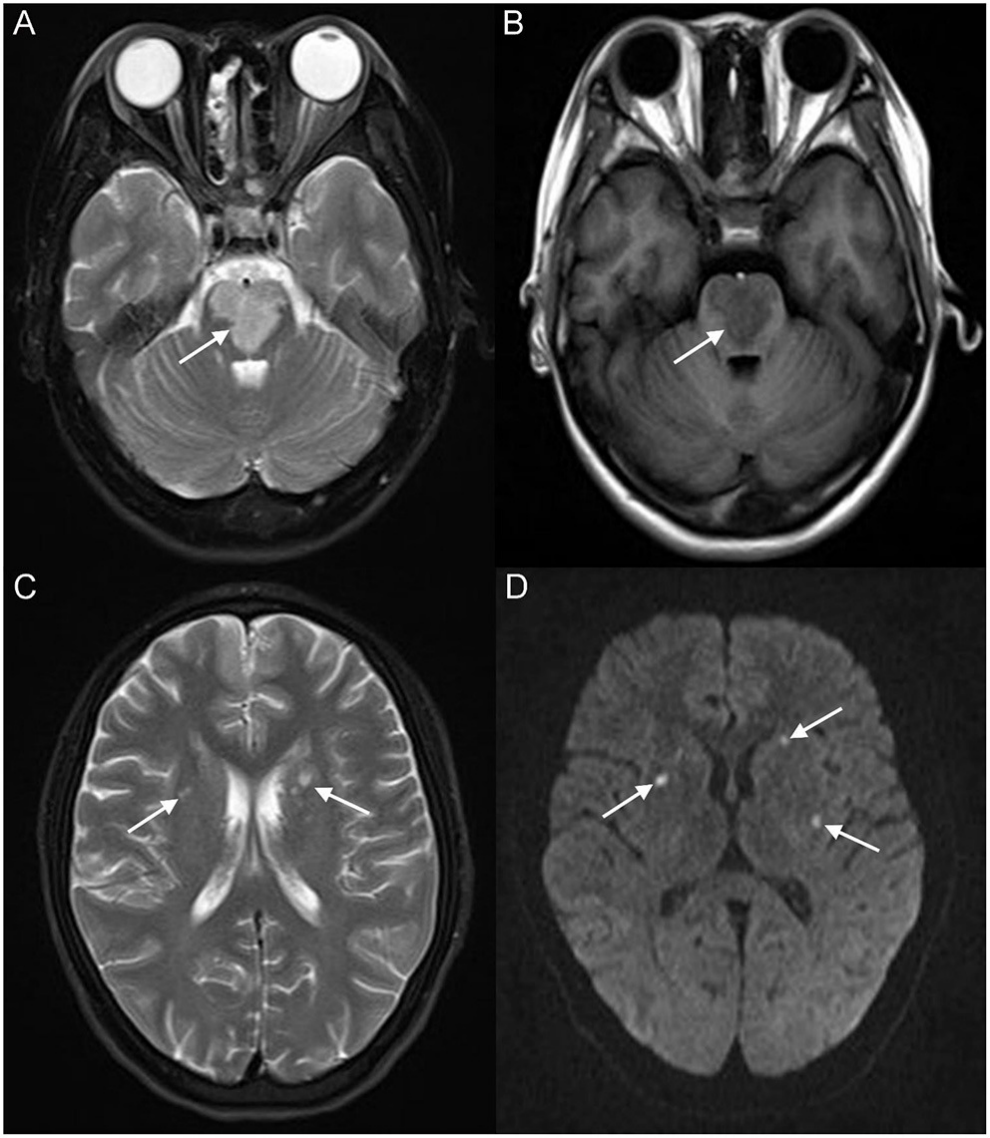
Brain magnetic resonance imaging (MRI) revealed extensive hyperintensity on T_2_-weighted imaging and hypointensity on T_1_-weighted imaging of the bilateral parts of the pons (white arrow) **(A,B)**; a patchy hyperintensity on T_2_-weighted imaging of the bilateral basal ganglia, bilateral centrum semiovale (white arrow) **(C)**, and right frontoparietal lobe, which presented a high signal on diffusion-weighted imaging (DWI) (white arrow) **(D)**.

**Figure 4:**
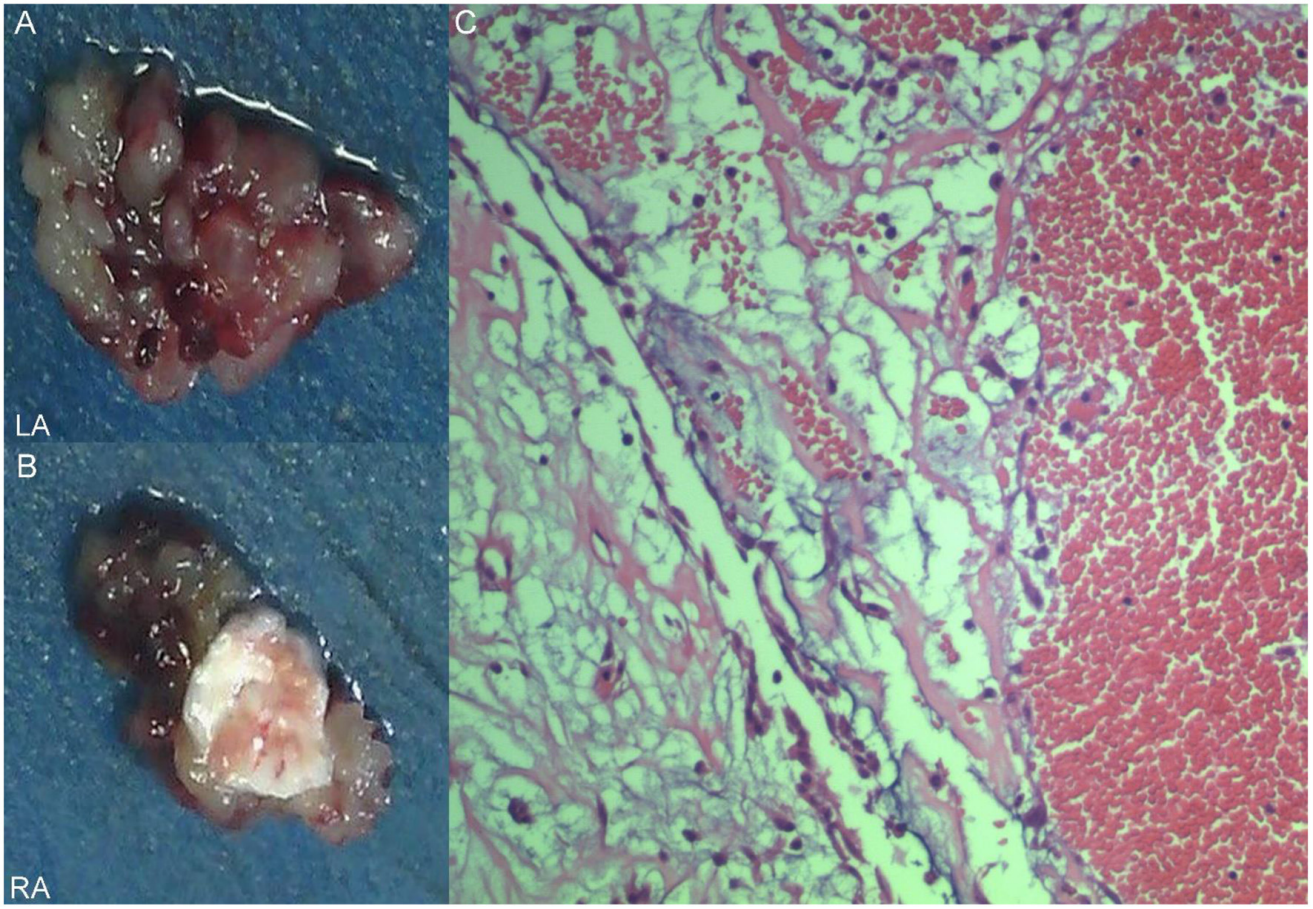
Excised villous biatrial atrial myxoma **(A,B)** and histology of atrial myxoma. Acid-mucopolysaccharide matrix with characteristic astrocytes and spindle cells which have ovoid nuclei and are surrounded by thin-walled capillaries (Hematoxylin and Eosin, × 200) **(C)**.

A 17-year-old girl presented to our hospital on August 22, 2021 with clouding of consciousness for more than 3 days. At 3+days prior, the patient was unable to speak the patient was unable to speak when called softly and unable to open the right eye, combined with involuntary movements of the limbs and incontinence. The patient visited the local hospital immediately, where relevant tests were performed, suggesting intracranial lesions. Since the specific treatment measures were unavailable at the previous hospital, she was transferred to our hospital for further treatment. There was no history of trauma or familial genetic diseases, such as high blood pressure and diabetes. Physical examination revealed a body temperature of 37.0°C, heart rate of 110 bpm, regular heart rhythm, blood pressure of 113/77 mmHg, and no pathological murmurs in the valve region; pulmonary auscultation revealed coarse rales in the entire lung. The pupils were equal, round, and pupillary light reflexes were delayed. Both lower limbs exhibited hypertonia and hyperreflexia of the knee and tendon reflexes. Laboratory examinations revealed the following levels (normal range): coagulation function test showed D-dimer was 0.57 mg/L (< 0.5 mg/L) and fibrinogen was 5.82 g/L (2.00–4.00 g/L). Routine blood tests showed that the absolute value of neutrophils was 7.38 × 10^9^/L (1.8 × 10^9^/L−6.3 × 10^9^/L). Infection-related markers showed that the hypersensitive C-reactive protein level was 111.563 mg/L (0.068–8.200 mg/L). Creatine kinase, α-hydroxybutyrate dehydrogenase, and lactate dehydrogenase levels were 148 U/L (26–140 U/L), 218 U/L (90–180 U/L), and 295 U/L (140–271 U/L), respectively. Computed tomography pulmonary angiography (CTPA) detected filling defects in the right atrium, left atrium, and left lower pulmonary basilar artery (Figure 1), and a diagnosis of Pulmonary embolism was made. Transthoracic echocardiography (TTE) revealed irregular iso-echoic masses in the bilateral atrium that were likely myxomas, given their location and appearance in a young patient; the myxoma in the left atrium measured approximately 38 × 21 mm, and it was attached to the junction of the lower part of the interatrial septum (IAS) and the root of the anterior mitral leaflet; in the right atrium it measured approximately 51 × 27 mm, and it was attached to the lower part of the IAS (Figure 2A). These masses resulted in the acceleration of the tricuspid valve antegrade flow (Figure 2B). Craniocerebral computed tomography (CT) showed extensive hypodensity in the bilateral parts of the pons and patchy hypodensity in the left corona radiata area and bilateral basal ganglia areas. Magnetic resonance imaging (MRI) also revealed extensive hypointensity on T_1_-weighted imaging and hyperintensity on T_2_-weighted imaging in the bilateral parts of the pons, and a patchy hyperintensity on T_2_-weighted imaging in the bilateral basal ganglia areas; bilateral centrum semiovale; and right frontoparietal lobe, which presented as high signal on diffusion-weighted imaging (DWI) (Figure 3). This confirmed the diagnosis of brain ischemia. To prevent thrombosis, low molecular weight heparin calcium (subcutaneous injection, 0.4 ml/12 h) was commenced on the second day of admission and continued until discharge. The patient underwent successful removal surgery for a biatrial myxoma. The masses were sent for histological examination, which confirmed the presence of a myxomatous matrix containing myxoma cells (Figure 4). The patient recovered uneventfully and was discharged 9 days after the procedure. The patient has been followed-up postoperatively for over 2.5 months, and limb motor function of the patient has recovered to some degree, but there is intellectual and cognitive decline.

## Literature Review

**Figure 5:**
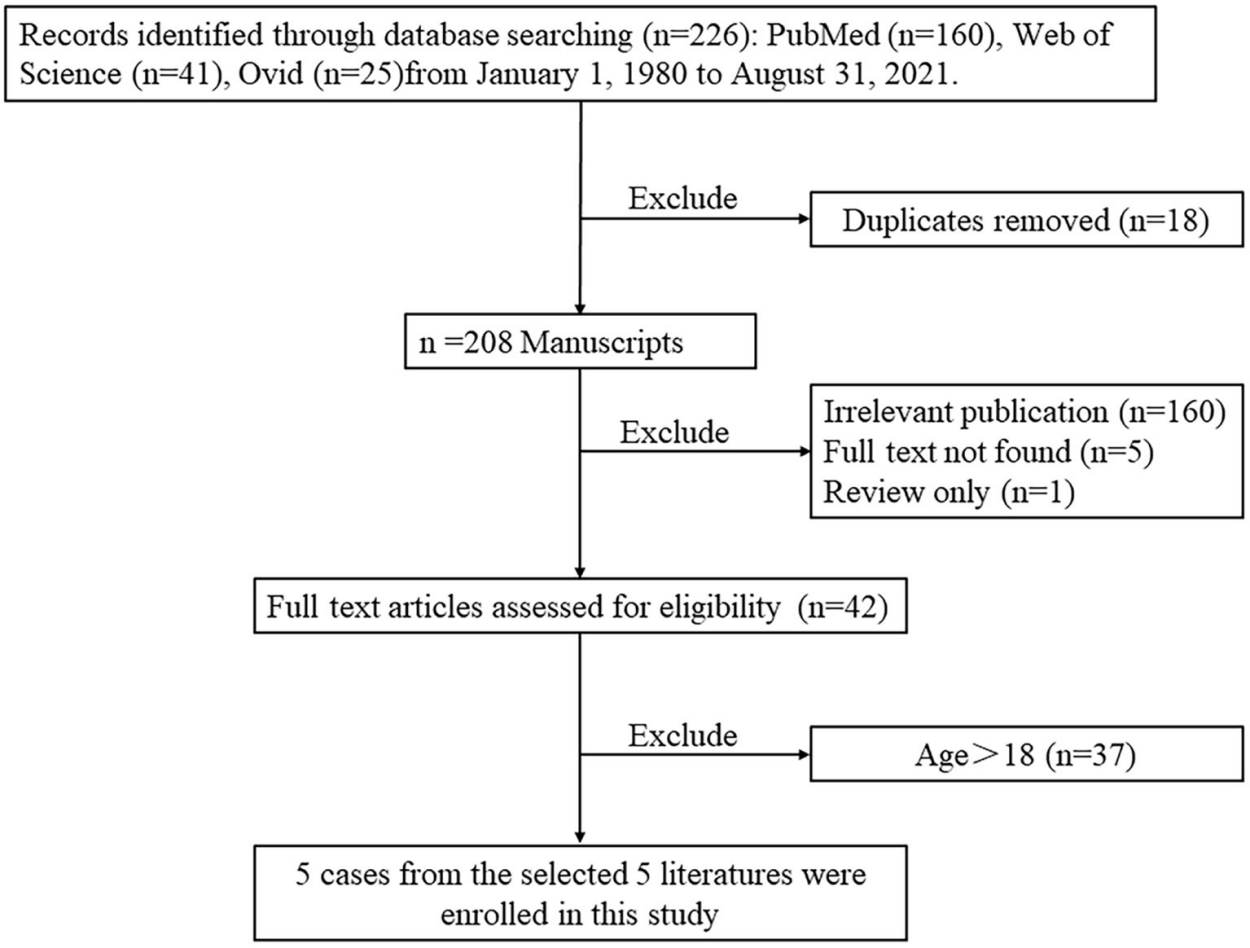
The flow chart of the literature screening process for biatrial myxoma in children and adolescent patients.

In order to present the literature review, case reports of all biatrial myxoma among child and adolescent patients in the English language were searched from the PubMed, Web of Science, and Ovid databases, dating between January 1, 1980 and August 31, 2021. Key words were used for the search, which included “biatrial myxoma,” “bilateral atrial myxoma,” “bilateral Atrial myxomas,” “pulmonary embolism,” and “cerebral embolism.” The flow chart of the literature screening process is presented in Figure 5. A total of five articles involving five cases were included in the analysis. For each case, we documented the first author, publication year, and the patient's age, sex, presentation, tumor size, image features, operation, outcome, and follow-up results (Table 1).

**Table 1:** The cases of biatrial. myxoma in children and adolescent patients from the literature review.

**Case (No.)**	**References**	**Sex**	**Age**	**Presentation**	**Tumor size (mm)**		**Image feature**	**Operation**	**Outcome**	**Follow-up (months)**
					**LA**	**RA**				
1	Hanly et al., (4)	F	14	Bilateral pleuritic chest pain/loss of energy	NM	NM	A-mode: sonic mass in the left atrium, and right atrium.	Yes	Discharged	16
2	Deshpande et al., (5)	F	18	Congestive heart failure	70 ×45 ×25	55 ×50 ×30	TTE: biatrial cardiac masses.	Yes	Discharged	24
3	Cilliers et‘al. (6)	M	8	Sudden onset of shortness of breath	39 ×26	6 ×38	X ray: large globular heart. TTE: large pedunculated biatrial masses.	Yes	Discharged	-
4	Mahilmaran et al., (7)	M	12	Fatigability, swelling of the legs, facial puffiness, abdominal distention, progressive breathlessness	30 ×19	80 ×40	TTE: an 8 ×4 cm myxoma arising from the right atrium, another pedunculated myxoma, 3 ×1.9 cm, was seen in the left atrium.	Yes	Discharged	24
5	Ananthanarayanan et al., (8)	M	14	Transient ischemic attack	60 ×40	40 ×30	TTE: biatrial myxoma attached to the interatrial septum.	Yes	Discharged	-
6	PC	F	17	Loss of consciousness	38 ×21	51 ×27	TTE: irregular iso-echoic masses in bilateral atrium. CTPA: filling defects in right atrium, left atrium, and left lower pulmonary basilar artery. Brain MRI: extensive hyperintensity on T2-weighted imaging and hypointensity on T1-weighted imaging of the bilateral parts of the pons.	Yes	Discharged	2.5

*PC, present case; F, female; M, male; LA, left atrium; RA, right atrium; NM, not mention; TTE, Transthoracic echocardiography; CTPA, computed tomography pulmonary angiography; MRI, magnetic resonance imaging*.

According to the literature, from January 1980 to August 2021, only 44 cases of biatrial myxoma have been reported. Our results revealed a predominance of biatrial myxoma between 40 and 65 years of age (52.2% of the cases), however, only six cases have been reported for patients of 18 years or less (about 13.6%; including of our case) of biatrial myxoma, comprising three girls and three boys, with a female to male ratio of 1:1. Remarkably, the presentation of these patients was predominantly associated with embolic events, such as pulmonary embolism and cerebral embolism. However, all patients with pulmonary and cerebral embolism had only one or the other, and the occurrence of both pulmonary and cerebral embolism simultaneously has not been reported. Fortunately, most patients were discharged after surgical operations, suggesting that biatrial myxoma has a relatively good prognosis.

## Discussion and Conclusions

In this case report, we present the case of a 17-year-old girl with biatrial myxoma, which is a rare, surgically correctable underlying cause of stroke and acute pulmonary embolism in a young patient. According to the clinical presentations and the results of echocardiography, clinicians suspected that the biatrial mass was a cardiac myxoma. As mentioned in the literature, cardiac myxoma is the most frequent benign tumor of primary cardiac tumors, accounting for more than 50% of primary cardiac tumors in adults, but only 5% occur in adolescents (6) and the incidence of cardiac myxoma is approximately 0.0008–0.015% (9). In addition, approximately 75% of cardiac myxomas involve the left atrium and approximately 20% of them are found in the right atrium, and cases of myxoma arising in the bilateral atrium are extremely rare, accounting for only 2.5% of the total incidence (10, 11). According to the literature review, only six cases of biatrial myxoma have been reported before (including our case) in pediatric patients. Moreover, only our patient had a biatrial myxoma complicated by pulmonary embolism and cerebral embolism.

The clinical manifestations and symptoms of cardiac myxomas vary and are not specific and can be divided into the following three groups: first, obstructive manifestations occur most frequently in approximately 50% of patients with atrial myxoma, including dizziness and dyspnea; second, embolization symptoms of myxoma, which affect more than one-third of patients with cardiac myxoma, and the presentations depend on the location of cardiac myxoma, such as pulmonary hypertension, chest pain, and severe headache; third, systemic symptoms, such as palpitation, fatigue, and fever, occur in approximately 58% of patients with cardiac myxoma (12, 13). However, patients with cardiac myxoma can also be asymptomatic (14). In our review, the most frequent symptom of the patients was dyspnea; subsequently, syncope and palpitations were also common.

Cardiac myxoma may cause embolic events during tumor tissue shedding, and cases of obstruction are relatively common in cardiac myxomas, and the morbidity rate of embolism is approximately 30–40% (14). Neurologic events are the most common embolic events followed by systemic embolic events, typically occurring at a rate of 42 and 29%, respectively. However, pulmonary embolic events are rare (15). Pulmonary embolism and cerebral stroke are uncommon but extremely significant complications of cardiac myxoma, with the risk of embolic events in cases of cardiac myxoma associated with the mass's appearance; typically, villous myxomas are more likely to cause embolism (16). Acute embolic stroke occurs when shedding tumor tissue reaches the cerebrovascular system, and it is often associated with high rates of mortality and disability, which poses a great risk to the life of the patients. Atrial myxomas have become a potential source of emboli; therefore, the primary presentation of many patients with a clinical history of myxomas is stroke (17, 18). In our case, the primary reason for the patient to go to the hospital for treatment was unconsciousness. Brain MRI revealed territorial cerebral infarction in the bilateral parts of the pons and small area cerebral infarction in the bilateral basal ganglia areas, bilateral centrum semiovale, and right frontoparietal lobe.

Pulmonary embolic events are rare, but when they occur, it is important to identify the source of the embolus, and in previous reviews of the literature, the majority of emboli originated from deep venous thrombosis (DVT), accounting for about 50–70%. In addition to DVT, cancer-related emboli are also quite common (19). Clinically significant embolic events are uncommon in patients with atrial myxoma. However, in cases of right atrial or right ventricular myxoma, embolectomy of tumor fragments into the pulmonary vasculature with subsequent pulmonary hypertension has been reported (14). In the early stages of pulmonary embolism, the clinical symptoms and imaging manifestations are atypical, and it is easy to misdiagnose pulmonary embolism as pneumonia; however, the role of non-contrast chest CT scans in the diagnosis of pulmonary embolism is limited; currently, the preferred technique for the diagnosis of pulmonary embolism is CTPA, which can detect filling defects in the pulmonary circulation and help to confirm the diagnosis of pulmonary embolism, and it is clinically important to assess the severity of a patient's pulmonary embolism based on the results of CTPA (20). In our case, infiltrates were seen in the posterior basal segment of the left lower lung lobe on non-contrast chest CT and the patient was diagnosed with pneumonia; subsequent CTPA clearly showed the infiltrates to be filling defects of the pulmonary artery.

Imaging such as CT, MRI, TTE, and CTPA play an important role in the preoperative diagnosis of cardiac myxoma. Transthoracic echocardiography can reveal the location of the tumor attachment and the appearance features of the mass. Computed tomography pulmonary angiography is a useful tool that shows the location, size, and appearance of pulmonary emboli. Imaging approaches can play a key role in the diagnosis of symptomatic or asymptomatic cases. We recommend non-invasive imaging examinations for preoperative diagnosis of patients with cardiac myxoma. According to the results of these examinations, the patients underwent surgery and the mass was totally resected, and their operation was successful.

Once the correct diagnosis has been validated, an effective treatment strategy should be determined. Surgical excision of the cardiac myxoma is the principal treatment modality and should be considered as the preferred option (21). Prompt surgical treatment is indicated to decrease the risk of complications and sudden death. However, our case represents a very complex situation of biatrial myxoma complicated by pulmonary embolism and cerebral embolism, and therefore, careful attention should be paid to certain perioperative details to achieve a predictable and successful surgical outcome.

Clinically, cases of cardiac myxoma are common; however, cases of bilateral myxoma combined with pulmonary embolism and cerebral infarction are rare. The clinical symptoms of myxoma are complex and variable, and patients may even be asymptomatic, based on whether valves are obstructed by cardiac myxoma and occurrence of systemic embolism and secondary symptoms. Computed tomography, MRI, TTE, and other imaging methods can effectively assist in making a definitive diagnosis and provide a guarantee for the treatment and prognosis of patients with myxoma. Once a diagnosis is established, surgical management is the primary treatment.

## Data Availability Statement

The original contributions presented in the study are included in the article/supplementary material, further inquiries can be directed to the corresponding author/s.

## Ethics Statement

The studies involving human participants were reviewed and approved by the Ethics Committee of The Affiliated Hospital of Zunyi Medical University. Written informed consent to participate in this study was provided by the participants' legal guardian/next of kin. Written informed consent was obtained from the individual(s), and minor(s)' legal guardian/next of kin, for the publication of any potentially identifiable images or data included in this article.

## Author Contributions

HR and GC: manuscript writing. JH and FL: manuscript revision. YH and JL: collection of data or analysis. HL and TZ: conception and critical review. All authors contributed to the article and approved the submitted version.

## Conflict of Interest

The authors declare that the research was conducted in the absence of any commercial or financial relationships that could be construed as a potential conflict of interest.

## Publisher's Note

All claims expressed in this article are solely those of the authors and do not necessarily represent those of their affiliated organizations, or those of the publisher, the editors and the reviewers. Any product that may be evaluated in this article, or claim that may be made by its manufacturer, is not guaranteed or endorsed by the publisher.
